# Discrete states and carrier-phonon scattering in quantum dot population dynamics

**DOI:** 10.1038/srep08267

**Published:** 2015-02-05

**Authors:** Minh Tan Man, Hong Seok Lee

**Affiliations:** 1 Department of Physics, Chonbuk National University, Jeonju 561-756, Republic of Korea

## Abstract

The influence of the growth conditions of multilayer CdTe/ZnTe quantum dots (QDs) on Si substrate upon their carrier dynamics is studied using intensity integration and broadening photoluminescence. The unusual temperature dependence of the line broadening is explained using a model for interband transitions that involves a lowest discrete electronic state (1S_e_) with different discrete hole states (1S_3/2_ and 2S_3/2_) and a 1P transition. These transitions are expected to play a critical role in both the thermally activated energy and the line broadening of the QDs. We also demonstrate that a thermally activated transition between two different states occurs with band low-temperature quenching, with values separated by 5.8–16 meV. The main nonradiative process is thermal escape assisted by carrier scattering via emission of longitudinal phonons through the hole states at high temperature, with an average energy of 19.3–20.2 meV.

Quantum dots (QDs) are interesting not only for their fundamental physics, but are also important for applications in optoelectronics and emerging electronic devices. The key characteristics of QDs and their suitability for use in distributed device active layers and discrete devices are heavily dependent on their growth conditions, which strongly influence dot size, uniformity, and composition. These device structures are typically grown on GaAs substrates due to the small lattice mismatch. However, the use of Si substrates has many advantages: they are cheaper and more widely available than compound semiconductor substrates, and Si technologies offer the potential for fabrication of very-large-area and high-quality products^1^. To make QDs suitable for use in practical applications, it is essential to reduce their sizes to increase the density of their surface distribution and ordering. Multilayer structures with spatially ordered QDs are characterized by highly homogeneous sizes and shapes; furthermore, the vertical alignment throughout subsequent layers can be engineered to be nearly perfect, although the lateral ordering tendency has been found to be much less pronounced. Accordingly, an understanding of carrier dynamics and time-resolved photoluminescence (PL), as well as precise control of shape and size distributions of QDs, are expected to increase opportunities for their utilization^2^,^3^ and to play vital roles in deterministic physical performance metrics that will improve optics and electronic devices. In addition, because the discrete structure of energy and density of states arise mainly from QDs' δ-functions, the carrier dynamics and energy relaxation in QDs are mainly expected to differ qualitatively from those in bulk materials. So far, several important mechanisms for explaining the unique evolution of PL spectra in QDs have been taken into account, including radiative relaxation energy transfer between dots of different dimensions^4^, Auger recombination scattering^5^, thermal escape from the dot^6^,^7^, and trapping in surface and/or defect states^8^,^9^. However, the mechanisms for carrier trapping and thermally activated transitions between different states, as well as the origin of trapping at defect sites, are not yet understood.

In this work, we investigated the carrier dynamics in multilayer CdTe/ZnTe QDs on Si substrate with various numbers of stacked dot layers. Analysis of the PL spectra as a function of temperature in conditions of low excitation power indicated that the confinement energy in the QDs was nearly independent of their temperature. Herein we show that interband transitions, which involve a lowest discrete electronic state (1S_e_) with different discrete hole states (1S_3/2_ and 2S_3/2_) and a 1P transition, are expected to play a critical role in both thermally activated energy and the broadening of PL spectra of the CdTe QDs. We demonstrate that the thermally activated transition between two different states occurs due to band low-temperature quenching with values separated by 5.8–16 meV, while the main nonradiative process is thermal escape assisted by carrier scattering via emission of longitudinal phonons through the hole states. This emission occurs at high temperature with an average energy of 19.3–20.2 meV.

## Results

**Figure 1:**
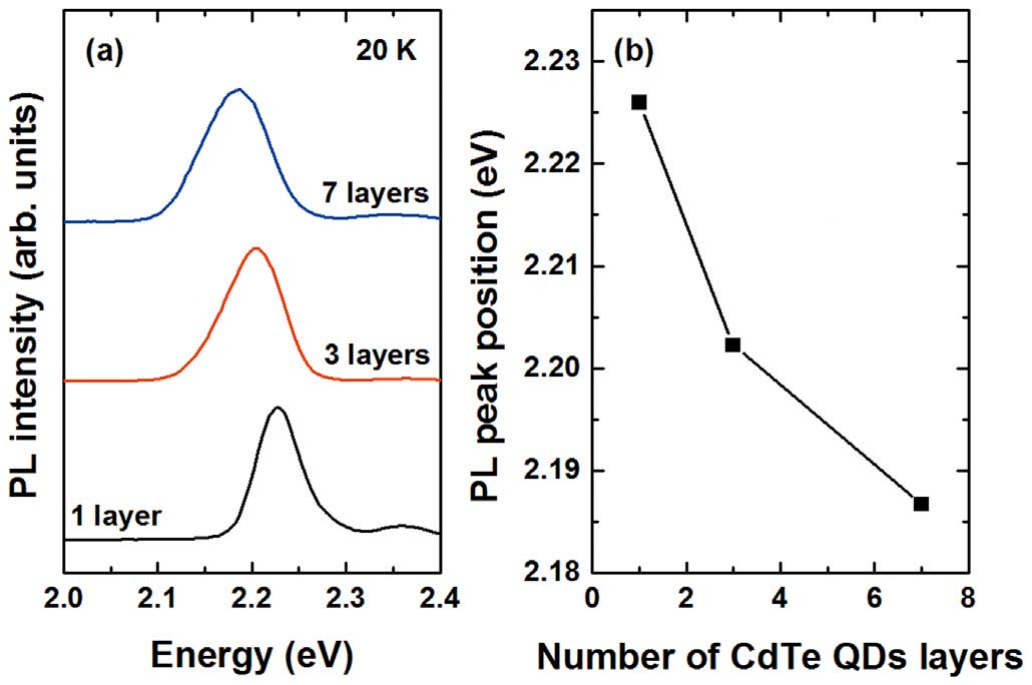
(a) PL spectra, at 20 K, of the one, three, and seven layer structures under low excitation power. (b) PL peak position as a function of the number of stacked dot layers for multilayer CdTe/ZnTe QDs on Si substrate.

PL spectra of the one, three, and seven layer QD samples, taken at 20 K, are shown in Fig. 1(a). We assumed that all photoexcited electron–hole pairs were effectively captured in the QDs, and assumed a laser excitation density (*I*_0_) of ~1 mW, an excitation photon energy of 3.098 eV, and a laser repetition rate equal to 76 MHz to give a photon fluence <*j_p_*> of ~3 × 10^11^ photons cm^−2^ per laser pulse. With the density of the CdTe QDs being ~2–5 × 10^10^ cm^−2^ and the spot diameter being ~100 μm, the average number of excited pairs in each dot is given by <*N*_0_> = *j_p_σ*_0_, where *σ*_0_ is the absorption cross section of a dot^10^. This result, <*N*_0_>, is approximately 0.2, which is small enough to neglect Auger scattering^11^. Under this excitation power (1 mW), the photoexcited electron–hole pairs would occupy the ground state of the QD at low temperature. The dominant peaks shown in Fig. 1(a) correspond to the exciton transition from the ground-state electronic subband to the ground-state heavy-hole band (*E*_1_*-HH*_1_) in the CdTe/ZnTe QDs. The single-layer sample possessed the highest-energy ground state of 2.23 eV; the three and seven layer samples had ground states of 2.2 eV and 2.18 eV, respectively. Figure 1(b) shows the peak position and full width at half maximum (FWHM) of the *E*_1_*-HH*_1_ transition for the multilayer CdTe/ZnTe QDs on a Si substrate as a function of the number of stacked dot layers. The redshift in the PL peak position for the multilayer QDs with increasing stacked dot layers was attributed to an increase in the size of the QDs due to intermixing between the ZnTe barrier and the CdTe QDs^12^. The FWHM of the *E*_1_*-HH*_1_ transition for multilayer CdTe/ZnTe QDs increases with increasing CdTe thickness, owing to inhomogeneous broadening resulting from the size distribution of the QDs^13^.

**Figure 2:**
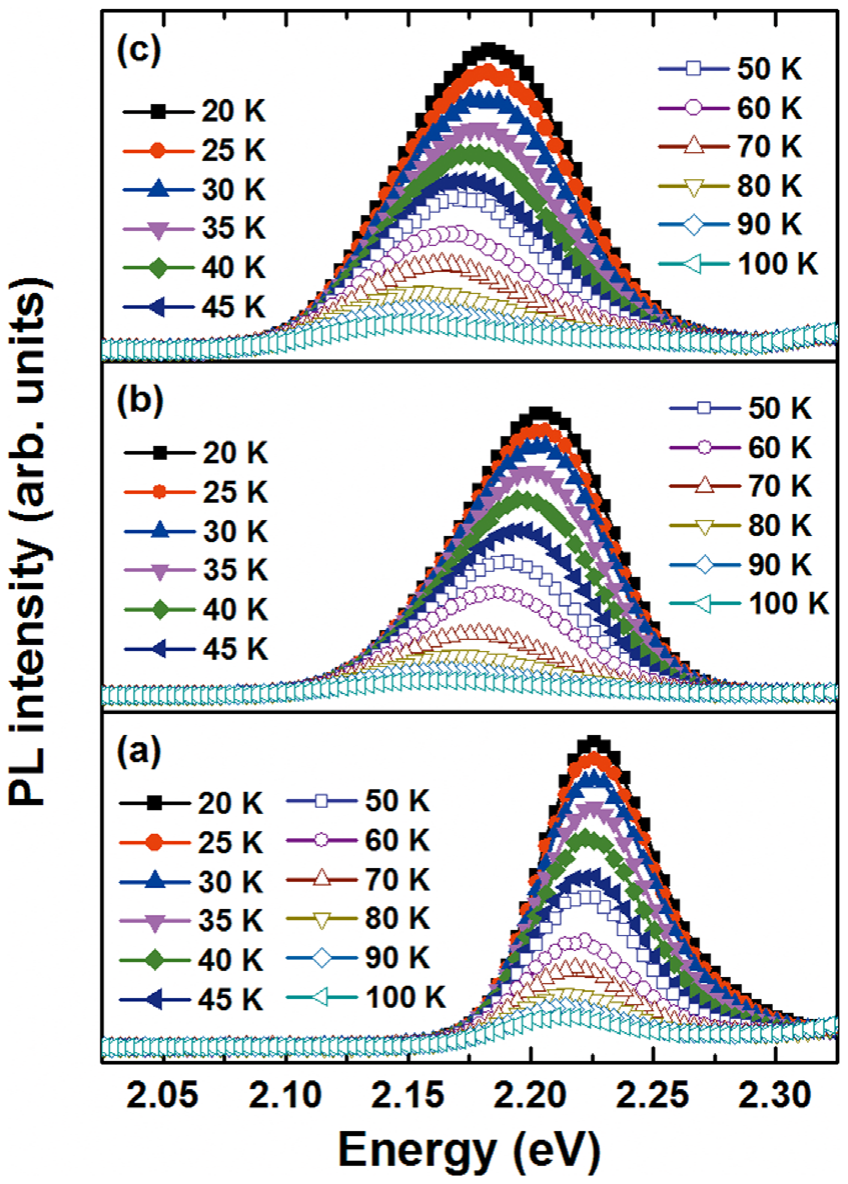
PL spectra as a function of temperature for QD samples of (a) one layer, (b) three layers, and (c) seven layers.

**Figure 3:**
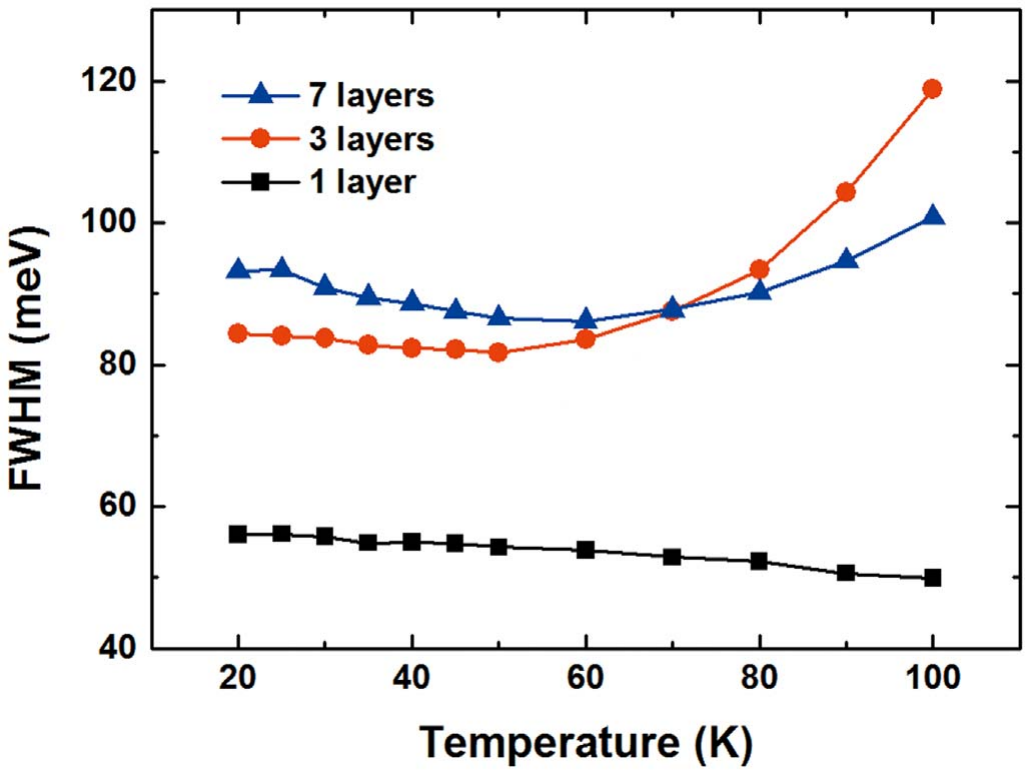
FWHM of the PL spectra for multilayer CdTe/ZnTe QDs on Si substrate.

**Figure 4:**
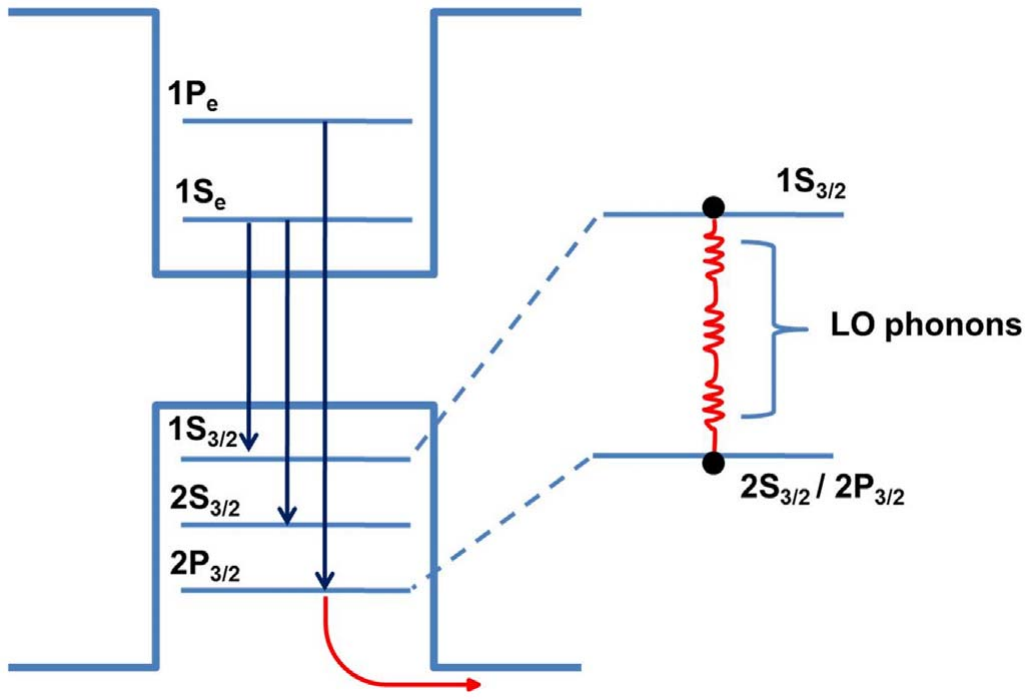
Schematic illustration of the different contributions to the joint density of states and thermal escape in CdTe/ZnTe QDs on Si substrate.

The carrier dynamics in PL emission from CdTe/ZnTe QDs have been investigated^14^, with the conclusion that PL emission originates primarily from the ground state. The main contributor among nonradiative processes is thermal escape from QDs, which is assisted by carrier scattering via emission of longitudinal phonons from excited QD states. Considering that size variation in the QD ensemble widens the PL emission spectrum, a phenomenon that is also referred to as inhomogeneous broadening, we also measured the temperature dependencies of the PL spectra of all samples at the excitation power of ~1 mW. Figure 2 shows PL spectra of QDs as a function of temperature. With decreasing temperature, we noted: a) a transition shift of the emission maximum to higher energies, b) variation broadening, and c) noticeable growth of the PL strength. The FWHM of the emission peak is displayed as a function of temperature in Fig. 3, in addition to a typical temperature dependence of the PL peak energies of self-assembled QDs, which represents the energy gap of average-sized dots. Observing the single-layer FWHM, it is seen that the broadening of the PL spectra reduces as temperature increases, accompanied by a redshift of emission energy with a slight sigmoid dependence on temperature. Significantly different temperature dependencies of the FWHM of the PL spectra were observed for the three- and seven-layer QDs. As the temperature was increased, anomalous reduction appeared within the temperature range from 20 to 50 K. The minimal FWHM of the PL spectra were found to be approximately 83 and 86 meV at 50 K for three and seven layers, respectively. However, the FWHM of PL spectra increased more rapidly with increasing temperature, becoming especially noticeable for temperatures above 50 K. These unusual temperature dependencies can be understood in terms of exciton localization by individual dots of various sizes. The main impact of size inhomogeneity is variation in the electrons' energy levels, and subsequently an inhomogeneous broadening of the ensemble properties. Repopulation explains the temperature dependence below 50 K: exciton localization is weakened because some carriers in the lower-energy state thermally populate the higher-energy state. The decrease of FWHM is related to the reduction of the emission energy distribution because the repopulation reduces the number of the sites contributing to the emission^15^. For the temperature range above 50 K, electron–phonon scattering becomes important in the observed increase of FWHM with increasing temperature. Both optical and acoustic phonons are relevant in this process. An important difference between these two phonon types is that the optical phonons have a relatively fixed frequency. The expression for homogeneous FWHM can be written as follows^16^:
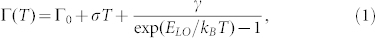
where *σ* is the coupling coefficient for couplings between excitons and acoustic phonons, *γ* is the coefficient accounting for the coupling between excitons and longitudinal-mode optical (LO) phonon coupling, and *E_LO_* is the LO phonon energy. We found the best fit values to be *σ* = 35 and 38 μeV/K, γ = 20.5 and 21.3 meV, and *E_LO_* = 19.8 and 19.3 meV for three and seven layers, respectively. The acoustic phonons, having lower energies (a few meV), played a dominant role at low temperature, with the observed magnitudes of the exciton–acoustic phonon coupling constants higher than the theoretical value estimated by Rubin *et al.* (~0.72 μeV for bulk CdTe^17^). This observation is consistent with the theoretical predictions of a strong increase of the coupling to acoustic phonons in nondimensional systems^7^. In general, the origins of carrier dynamics are commonly assigned to radiative relaxation, Auger nonradiative scattering, energy transfer between dots of different dimensions, thermal escape, and trapping in surface and defect states. To gain deeper insight into the nature of the variation of PL linewidth and decay times with respect to temperature across the entire temperature range, we modeled PL linewidth starting from the thermal redistribution of electrons and holes over the discrete states of a dot, without wetting layers. This model similar to and adapted from other models that have been demonstrated in literature^18^,^19^,^20^. A schematic of the model showing redistributions of electrons and holes separately is given in Fig. 4. We will consider heavy holes, which correspond to the lowest electron states and excited hole states. At low temperatures, the two lowest bands can be assigned to transitions involving the lowest electron state (1S_e_) and two different hole states (1S_3/2_ and 2S_3/2_), or equivalently, 1S[1S_3/2_(h)-1S_e_] and 2S[2S_3/2_(h)-1S_e_]. At higher temperatures (or higher energy), carriers occupy higher-lying energy levels, with the transition coupling the 1P electron state to the 1P_3/2_(h) hole state (1P transition). The value of <*N*_0_> is approximately 0.2 (<1), which is small enough to neglect the transition involving the 1S_e_ electron state and a hole state originating from the spin-orbit split-off band. We have also neglected the small contribution arising from wetting layer state energy. We recall that in the canonical ensemble, temperature is defined in terms of probability of occupation *P*(*E*) of the state of energy *E* through the relation 
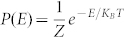
. Here, *Z* is the canonical partition function. It therefore follows that every state has an equal probability of occupation *P*(*E*) * = * 1/*Z* when the temperature is infinite. The sum over all the level occupation probabilities for the carriers in radiative process must be unity:
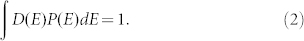


Setting the 1S_3/2_-state energy to correspond to the ground state, and setting the energy-level transition between two different states separated by localization energy to be Δ*E*, *D*(*E*), the carrier density of states, is then
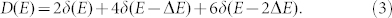


The terms of Eq. (3) represent the degeneracies of the first (1S_3/2_-1S_e_), second (2S_3/2_-1S_e_), and third (2P_3/2_-1P_e_) states, which are due to the spin degeneracy. We can determine the partition function *Z*(*T*):
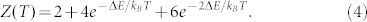


Then, the occupation probabilities of interband transitions can be obtained as follows:
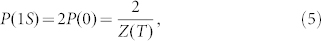

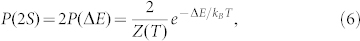

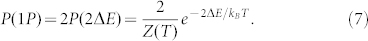


To calculate the total radiative lifetime, *τ_rad_*, we average over the accessible energy states:
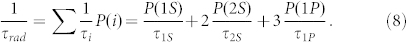


The radiative lifetime of the lowest transition (1S_3/2_-1S_e_) is now given by
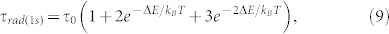
where *τ*_0_ is the radiative lifetime of the lowest transition (1S_3/2_-1S_e_) at 0 K. In a simple approach, we then consider different recombination rates of the QDs' ground state: discrete recombinations between electrons and holes in orbital channels with radiative lifetimes. The nonradiative recombination arises from thermal escape out of QDs, assisted by carrier scattering via LO phonons. With regard to thermal escape, the energy difference between the 1S_3/2_ and 2S_3/2_ hole states can be estimated simply as the two first absorption peaks, which correspond to the 1S_3/2_-1S_e_ and 2S_3/2_-1S_e_ transitions^21^,^22^,^23^. The thermal escape rate, *τ*_esc_, which involves hole states, is given by^24^ as
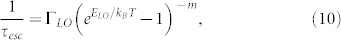
where Γ*_LO_* is a fitting parameter acting as a weight for the probability of carrier–phonon scattering, and *m* is the number of LO phonons involved in the process. Thus total decay time, *τ*, reads
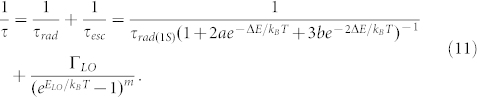
where *a* and *b* are fitting parameters related to the relative energy density of states.

## Discussion

**Figure 5:**
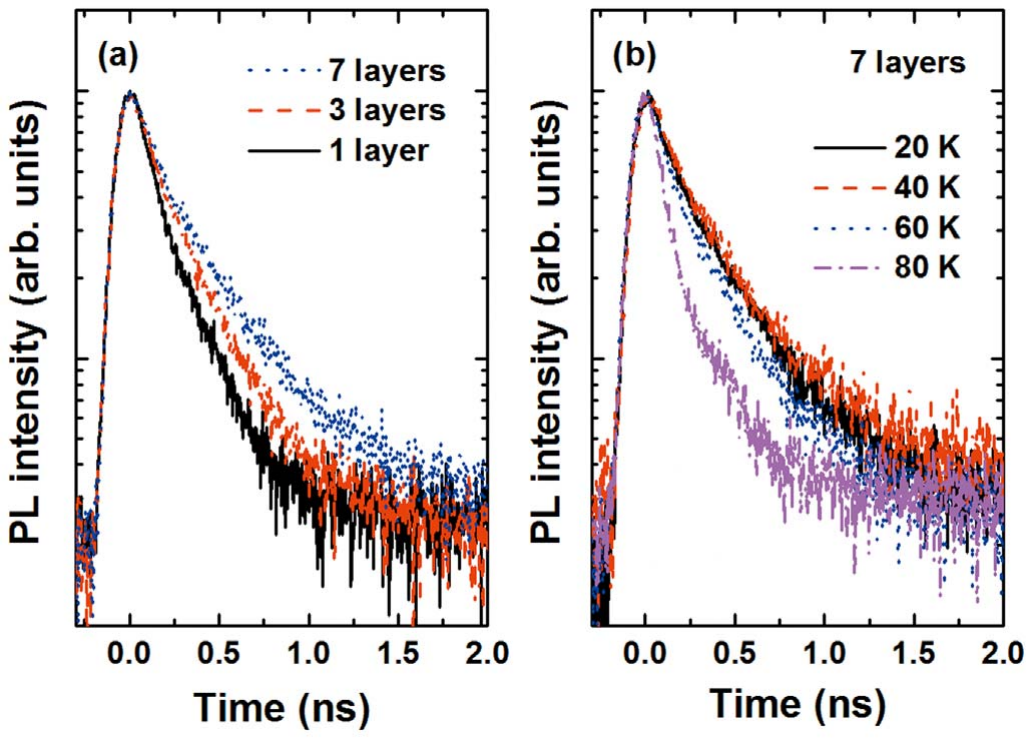
(a) Time-resolved PL spectra taken at 20 K of multilayer CdTe/ZnTe QDs on Si substrate. (b) Time-resolved PL spectra at several temperatures for QD sample of seven layers.

Fig. 5(a) shows the PL decay time obtained from experimental observations of all three samples under a low power density of 1 mW at 20 K. In order to extract the PL decay time, we performed reconvolution fitting with the IRF for the time-resolved PL results, which fit well with the biexponential function. As reported in literature, the fast component can be attributed to “bright” exciton decay and the slow component to “dark” exciton decay^25^. Hereafter, we focus only on the fast component, which resulted in decay times of 115, 165, and 197 ps for a single layer, three layers, and seven layers, respectively. The PL decay time became longer in QDs with greater numbers of stacked dot layers. This can be explained by the difference in coupling between electrons and holes induced by redistribution of carriers in interband transitions. The time-resolved PL spectra for seven layers measured at several temperatures are shown in Fig. 5(b).

**Figure 6:**
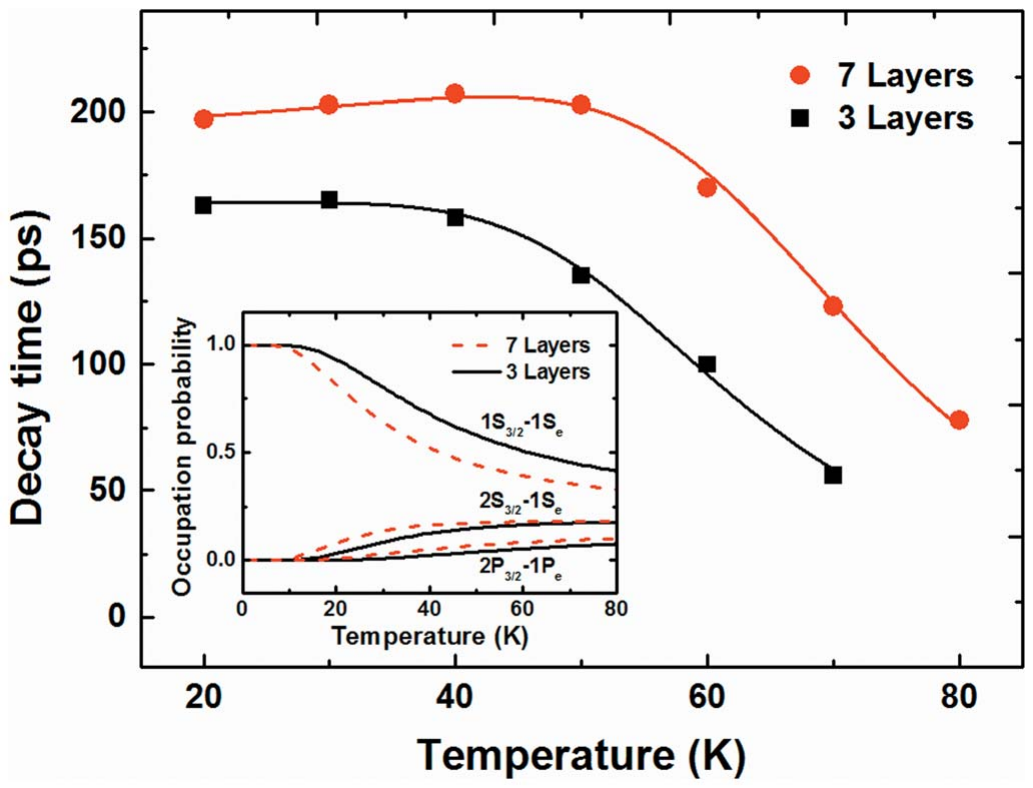
Theoretically estimated decay time of the lowest excitonic states as a function of temperature with experimental results for QD samples of three and seven layers. Inset: temperature dependence of the occupation probabilities of the electron and hole 1S, 2S, and 1P transitions.

Moreover, we compared our experimental data and calculations of PL decay time versus the temperature of three and seven layer samples, which are shown in Fig. 6. The fitting curves to Eq. (11) excellently reproduced the experimental data for Δ*E* = 9.1 meV, *E_LO_* = 19.7 meV, *m* = 1.75, and *τ_rad_*_(1*S*)_ = 164 ps for three layers; and Δ*E* = 6.1 meV, *E_LO_* = 19.3 meV, *m* = 2.0, and *τ_rad_*_(1*S*)_ = 199 ps for seven layers. To take into account these features, we calculated the temperature dependence of the occupation probabilities of the electron and hole 1S, 2S, and 1P transitions when the thermal escape process is taken into account (Inset in Fig. 6).

**Figure 7:**
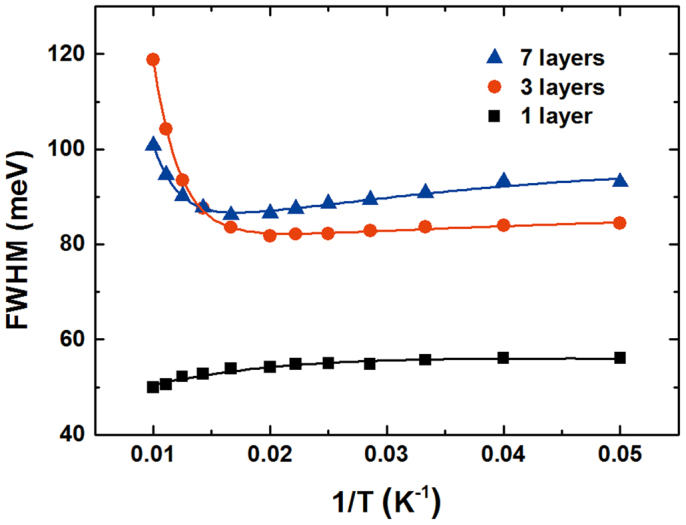
Measured (symbols) and calculated (solid lines) FWHM as a function of inverse temperature for the multilayer CdTe/ZnTe QDs on Si substrate.

Let us pay attention to PL linewidths with respect to temperature across the whole temperature range of all multilayer samples. The linewidth, Γ*_PL_*, is then determined by 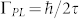 for various scattering mechanisms. It is given by
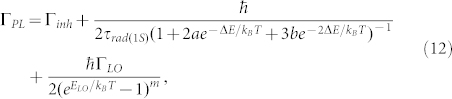
where Γ*_inh_* is the inhomogeneous broadening, which is temperature-independent and originates from inhomogeneities in size, shape, and configuration of the nanocrystals. The theoretically estimated FWHM of the lowest excitonic states for all samples are shown in Fig. 7 versus inverse temperature; Fig. 3 also shows experimental results. By fitting the above equation to the experimental data, Δ*E, E_LO_*, and *m* were determined to be 9.8 meV, 20 meV, and 2.2 for three layers; similarly, the respective values of 5.8 meV, 19.3 meV, and 2.2 were obtained for seven layers. Good overall agreement was obtained between theory and experiment. *E_LO_* and *m* of the carrier–phonon scattering of the single-layer sample were not evaluated; their origin is discussed below in terms of the radiative recombination process at low temperature. More precisely, the separated energy, Δ*E* = 16 meV, we calculated to be greater than other samples, suggesting a thermally activated transition between two different states. This suggested that the heavy hole states are filled when the thermal energy redistributes the carriers into the states, and that only the radiative recombination process is affected by thermal activation of the electrons.

**Figure 8:**
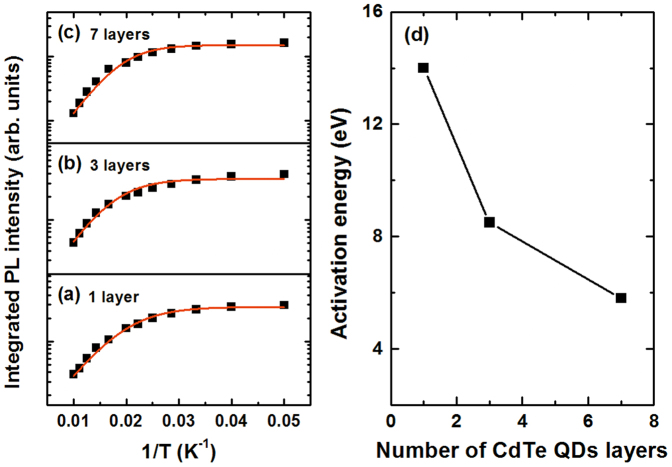
Integrated PL intensities as a function of temperature for the multilayer CdTe/ZnTe QDs on Si substrate; solid lines are the best fit curves with the interband transition and thermal escape process.

More clearly, to determine the difference in nonradiative processes affecting carrier relaxation, the peak integrated PL intensity of the QDs' emission was plotted as a function of the inverse temperature on a semi-logarithmic scale, allowing for the determination of separated energy of the thermal processes triggered at high temperatures (Fig. 8). To examine the PL intensity, we compared two PL intensity traces measured at high and low temperature. The integrated intensity was almost constant up to about 40 K, and decreased dramatically for temperatures higher than 40 K. In general, the intensity of the PL emitted per unit time for the carrier population *N* can be considered as an exponential decay over time:
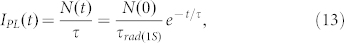
with *N*(0) as the initial carrier population and *τ* determined by Eq. (11). The temperature dependence of the integrated PL intensity is instead given by
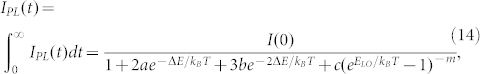
where *I*(0) is the integrated PL intensity at 0 K and *c* is a constant relating to the relative energy density of states. The solid curves in Fig. 8 represent the theoretical fitting based on Eq. (14). We found the best fit values to be Δ*E* = 8.5 meV*, E_LO_* *= * 20.2 meV, and *m* = 2 for three layers; and Δ*E* = 5.8 meV, *E_LO_* = 19.5 meV, and *m* = 2.2 for seven layers. For the single-layer sample, the best fit was Δ*E* = 15.8 meV; the parameters of the thermal escape process were not evaluated.

We observe that these best fit values, for all samples, were very similar to the values extracted from the PL linewidth analysis. The values of Δ*E* strongly decreased with increasing numbers of stacked dot layers. This separated energy involving carrier trapping in surface states is responsible for band low-temperature quenching. The importance of trapping by surface defects is expected to decrease with increasing uniformity in size, shape, and interdot spacing in the QD multilayers. However, defects in the ZnTe barrier are expected to play a vital role in the radiative recombination process because the carriers are delocalized on excited levels, allowing their wave functions to penetrate deeper into the barriers. Observation of multilongitudinal optical phonons suggested that the main nonradiative process at high temperatures was thermal escape. The observed best-fit values of the LO energy, *E_LO_*, were smaller than the bulk value of 24.5 meV^14^. Our result is consistent with the values of average phonon energies obtained for colloidal CdTe-QDs^21^ and self-assembled CdTe/ZnTe QDs^23^. We have accounted for the ability of such a configuration to confine the holes in the interband more effectively. Thus, the exciton binding energy is enhanced, and, conversely, the exciton-phonon coupling strength is suppressed. Furthermore, this additional outer layer can be used to increase the passivity of the surface states and thereby to reduce surface trapping.

In summary, we have studied the influence of the growth conditions of multilayer CdTe/ZnTe QDs upon their carrier dynamics, using PL spectra and PL lifetime measurements. The first effect is related to the redistribution of carriers in interband transitions, which are expected to play a critical role in both the thermally activated energy and the broadening of PL spectra of the multilayer CdTe QDs. The second effect is that thermally activated transitions between different states strongly decrease with an increasing number of stacked dot layers, which, involving carrier trapping in surface states, are responsible for band low-temperature quenching. The main nonradiative process is thermal escape assisted by carrier scattering via emission of LO phonons through the hole states at high temperature, with an average energy of 19.3–20.2 meV.

## Methods

### Sample structure

Several multilayer CdTe/ZnTe QDs with different stacked dot layers were grown on a Si (100) substrate using the following molecular beam epitaxy (MBE) and atomic layer epitaxy (ALE) procedures. First, the Si substrate was etched in a mixture of NH_4_F and HF (7:1) at room temperature for 1 min and then rinsed in deionized water. Immediately after this chemical cleaning process, the substrate was mounted on a molybdenum susceptor. Then, a 900-nm ZnTe buffer layer was grown on the Si substrate using MBE. Then, 2.5-monolayer (ML) CdTe QDs of one, three, and seven layers were grown using ALE. The CdTe QDs were separated by a 20-nm ZnTe spacer layer grown using MBE. The CdTe QDs were then capped with a 100-nm ZnTe layer grown using MBE. During the depositions of both the ZnTe and CdTe layers, the substrate was held at 320°C. The Zn and Te source temperatures used for the deposition of the ZnTe layer were 280 and 300°C, respectively; the Cd and Te source temperatures used for the deposition of the CdTe layer were 195 and 300°C, respectively. One cycle of ALE growth was implemented by means of an optimum growth process in which the Cd effusion cell was opened for 8 s and growth was interrupted for 1 s. Afterward, the Te effusion cell was opened for 8 s and growth was interrupted for 5 s. It should be noted that this interrupted process was adopted to improve the film quality by stabilizing positive and negative ions on the surface.

### Measurement techniques

Time-resolved PL decay curves were measured using a time-correlated single photon counting method. The excitation source produced 400-nm frequency-doubled femtosecond pulses from a 76-MHz mode-locked Ti:sapphire laser system, and the sample temperature was varied between 20 and 100 K using a He closed-cycle Displex refrigeration system. The PL was dispersed using a 15-cm monochromator and detected using a multichannel plate photomultiplier tube. A commercially available time-correlated single photon counting module (PicoHarp, PicoQuant GmbH) was used to obtain PL decay curves. The FWHM of the total instrument response function was less than 130 ps.

## Author Contributions

H.S.L. conceived and led the study. M.T.M. carried out the theoretical and numerical calculation. Both authors wrote and reviewed the manuscript.
